# Supplementary figures and images for: Apoptosis in idiopathic inflammatory myopathies with partial invasion; a role for CD8+ cytotoxic T cells?
Source: PLoS One. 2020 Sep 16;15(9):e0239176. doi: 10.1371/journal.pone.0239176 (PMC7494097; doi:10.1371/journal.pone.0239176)

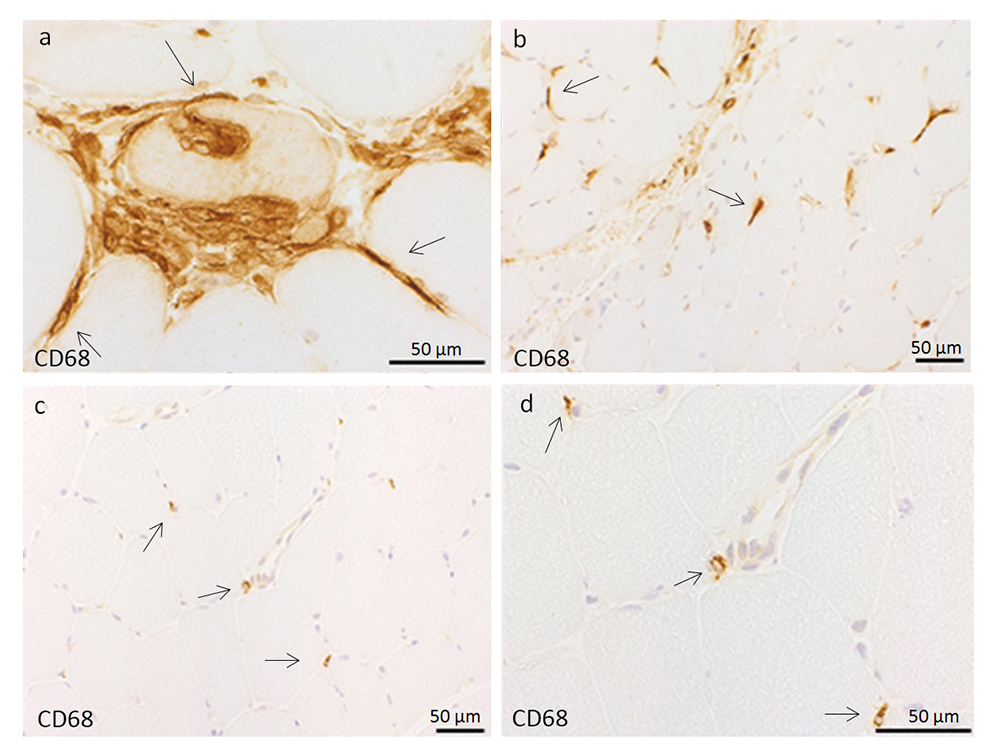

Supplement: S1 Fig — The stains of the CD68+ and the CD163+ cells in the cases with partial invasion (a) and dermatomyositis (b) indicate an expanded cytoplasm, compatible with a dendritic morphology, which is not seen in the non-inflammatory control cases (c and d [close-up]). (TIF) [file pone.0239176.s002.tif]

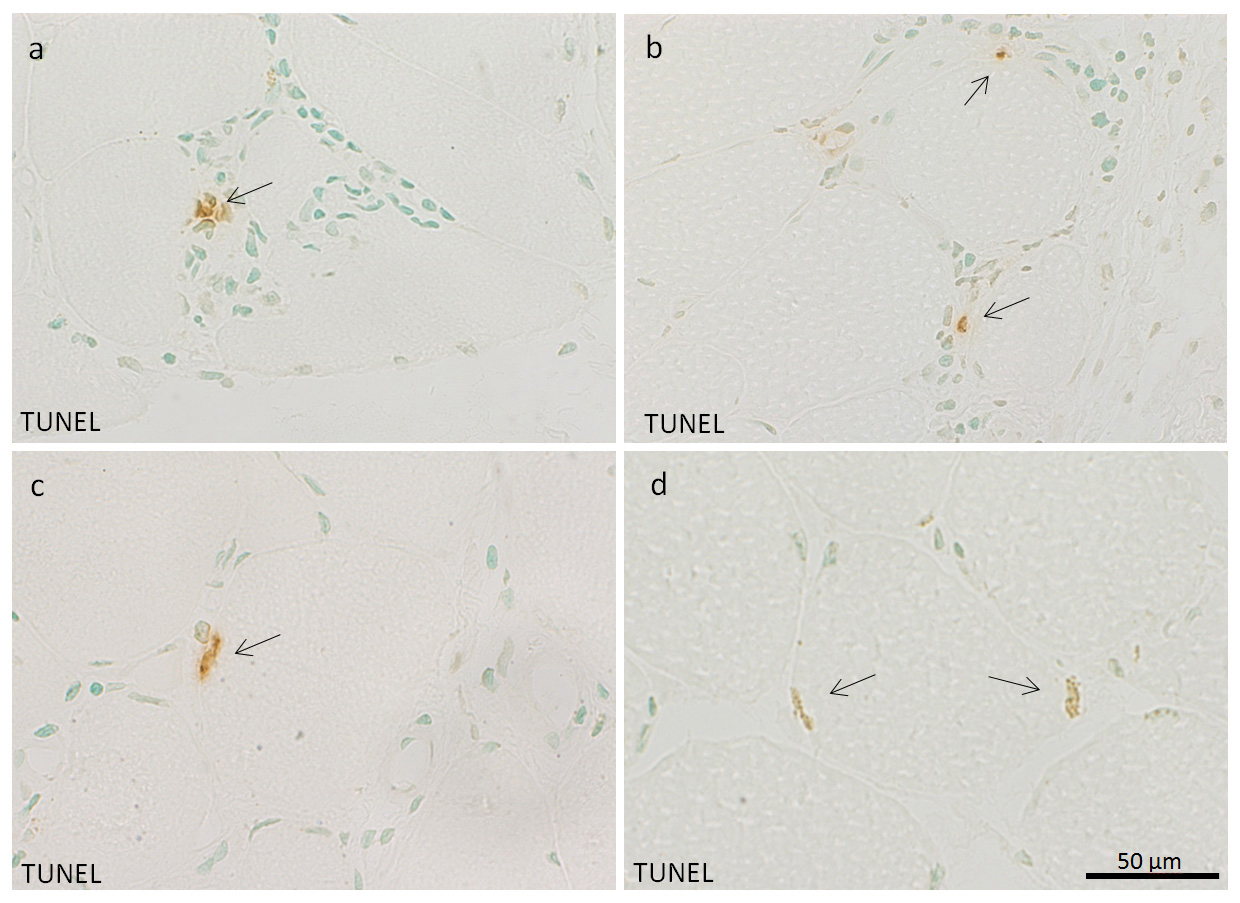

Supplement: S2 Fig — Two examples of TUNEL+ inflammatory cells (arrows) are shown, one close to a partial invasion with a branching appearance (a) and one in a case with dermatomyositis (b). When investigating the whole sections of the cases with partial invasion 45 TUNEL+ myonuclei were found, as judged by their morphological appearance and location, as exemplified here (c). One of the 3 cases in which 2 TUNEL+ myonuclei in the same fibre where found is shown (d). (TIF) [file pone.0239176.s003.tif]

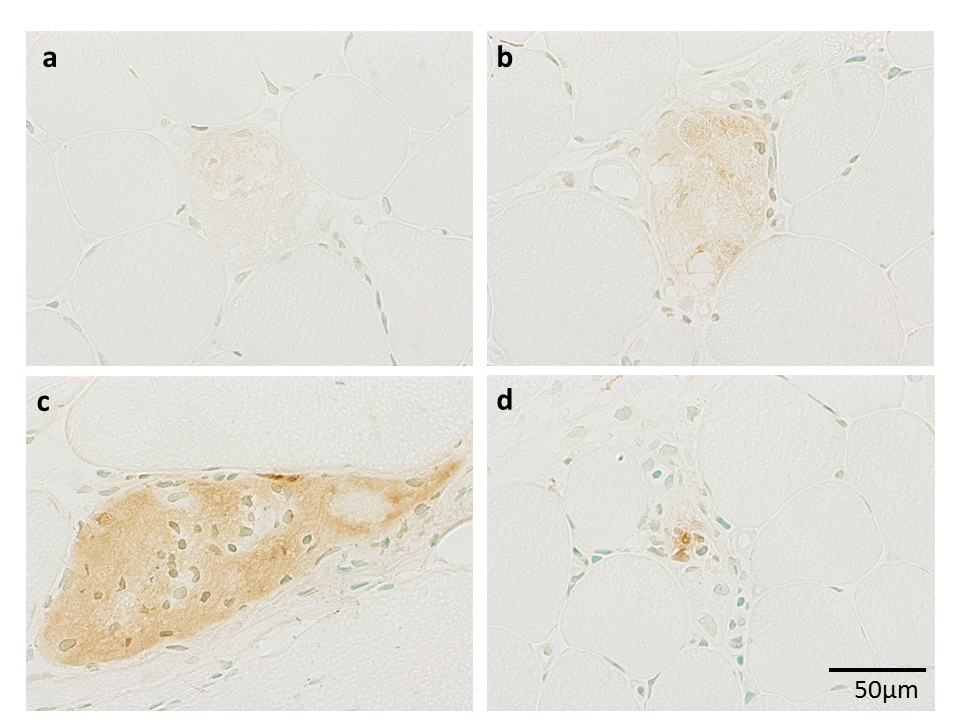

Supplement: S3 Fig — The vast majority of the necrotic fibres showed no TUNEL-stain (a). But rare necrotic fibres did show varying degrees of diffuse sarcoplasmic stain (b,c). The nuclei of some invading inflammatory cells stained TUNEL positive (d). (TIF) [file pone.0239176.s004.tif]
