# Supplementary material for: Apoptosis in idiopathic inflammatory myopathies with partial invasion; a role for CD8+ cytotoxic T cells?
Source: PLoS One. 2020 Sep 16;15(9):e0239176. doi: 10.1371/journal.pone.0239176 (PMC7494097; doi:10.1371/journal.pone.0239176)
Supplement: Attachment — Submitted filename: Comments reviewer.pdf [file pone.0239176.s005.pdf]

Olof Danielsson et al, analysed muscle biopsies from 10 IIM patients, 10 DM patients and 10 donors without muscle disease. They performed TUNEL assays and immunohistochemistry on muscle biopsies. The authors have four main conclusions :

1. IIM is associated with alteration in the expression of Bcl-2, FAS, HSP70, CD163 and CD68.
2. Apoptotic nuclei express MHC I but not FAS
3. Apoptotic nuclei are found almost exclusively in IIM with partial invasion
4. Majority of invading cells in partial invasions are CD8+ cytotoxic cells

Authors suggest that partial invasion and apoptosis are likely mediated by cytotoxic CD8 cells

Major comments :

1. Title of the manuscript is « Partial invasion and apoptosis in idiopathic inflammatory myopathies; parallel processes mediated by CD8+ cytotoxic T cells », nevertheless the authors have only 10 cases of IIM, including 3 IBM def (1 poss), 1 overlap def, PM….. Following the 119th ENMC classification, IIM also include IMNM and DM. Why is DM placed separately in a different group, why is there no IMNM case. Sample size is a little small to conclude.

2. Line 299 : *« In the DM-cases, 2 of the 4 TUNEL+ fibres 300 were found in a section which also contained a (sic) partial invasion.* ». How many DM patients have partial invasions.

3. Line 190 : « *IIM is associated with alteration in the expression of Bcl-2, FAS, HSP70 and of CD163/CD68 ratio* ». Authors could consider changing this sentence, as no significant differences in the expressions of FAS and HSP70 are observed between DM and controls (DM are IIM).

4. Table 1 : Authors show necrotic fibres in 4 out of 10 patients. Surprisingly necrotic fibres are not TUNEL+ fibres and vice versa. It has been described that TUNNEL assay does not discrimate apoptosis from necrosis (BETTINA GRASL-KRAU et al, HEPATOLOGY, May 1995). How can the authors explain these findings.

5. The lack of expression of FAS in apoptotic nuclei is indeed surprising. Nevertheless, considering the relatively low mean frequency of TUNEL+ myonuclei (0.22 per 100 fibres) and the presence of TUNEL+ fibres in only 5 out of 10 patients, it is possible that these TUNEL+ myonuclei do not have any pathological significance. A greater sample size could help make the results more robust.

6. Authors show increase frequency of FAS+ fibers in pi-patients compared to controls, nevetheless apoptotic fibers do not express FAS. How can the autors explain this, do non-apoptotic fibers express FAS.

7. Line 99 : control samples are young compared to pi-patients, mark difference in age distribution.

Minor comments :

1. Table 1 : Patient 7, change « IMB » to « IBM »

2. How can the authors confirm that partial invasion and apoptosis are mediated by CD8 cytotoxic cells. Can the authors show co-immunostaining of CD8 and granzyme B.

3. Line 231 : « *In addition to CD8+ cells, granzyme B+ cells were found in all invading infiltrates (Table 2).* » Table 2 shows Antibodies and immunohistochemical procedure, maybe the authors wanted to state Table 3 ?
